# An Interesting Lecture

**Published:** 1920-11-27

**Authors:** 


					An Interesting Lecture.
SheHielil V?*/' A!lll0IT' woman editor of tlie
"veftflhSSTr"iK'
aid of tli > p . vl ture 0,1 "Women and Flying, 11
the Lectin- I'/ n ^? eni HospitaJ. It will be held i'1
(near th 11 , the UPPer Holloway Baptist Church
Mfc, \hl ?SPlt?' ?" Frido* ^oember 3 at 8 **?
Loxlev V?H her fu'sM?ght on April 1914 (up the
famous vn u 1 SheffieId') i? a monoplane, with tbe
burn. ' In lgig1"0} P,Iot Mr' ("0W M"Jor) Harold BJft
R F p 1? Went at Coalaston with Captain
quentlv t ' 1 J,?'"0' one of the pilots who subf'
Dr pi , 77mca Vicftere-'Vimy aeroplane, with
aeri-n ^ T;S"Mit0hen' <*? th? Cairo to the C-P?
tio,, n, X^e '0"- M,as Abbott attended the first a*1?
1909 ee m? 1 "l K"?Ia"d at JJoncaster in Octobej
'ivint' !n,CO then hi,s bee? constantly writing about
tio,', 'Vdmisslo? to the lecture i* free, and a ooH?c-
evening. " ?f the h?8Pital wi" be during the

				

